# A health-conformant reading of the GDPR’s right not to be subject to automated decision-making

**DOI:** 10.1093/medlaw/fwae029

**Published:** 2024-08-12

**Authors:** Hannah B van Kolfschooten

**Affiliations:** Law Centre for Health and Life, Amsterdam Law School, University of Amsterdam, 1018 WV Amsterdam, The Netherlands

**Keywords:** Artificial Intelligence, automated decision-making, EU law, GDPR, healthcare, patients’, rights

## Abstract

As the use of Artificial Intelligence (AI) technologies in healthcare is expanding, patients in the European Union (EU) are increasingly subjected to automated medical decision-making. This development poses challenges to the protection of patients’ rights. A specific *patients’ right not to be subject to automated medical decision-making* is not considered part of the traditional portfolio of patients’ rights. The EU AI Act also does not contain such a right. The General Data Protection Regulation (GDPR) does, however, provide for the right ‘not to be subject to a decision based solely on automated processing’ in Article 22. At the same time, this provision has been severely critiqued in legal scholarship because of its lack of practical effectiveness. However, in December 2023, the Court of Justice of the EU first provided an interpretation of this right in C-634/21 (SCHUFA)—although in the context of credit scoring. Against this background, this article provides a critical analysis of the application of Article 22 GDPR to the medical context. The objective is to evaluate whether Article 22 GDPR may provide patients with the right to refuse automated medical decision-making. It proposes a health-conformant reading to strengthen patients’ rights in the EU.

## I. INTRODUCTION

The importance of Artificial Intelligence (AI) technologies in medical decision-making is steadily increasing and paves the way for the embedding of *automated medical decision-making* in regular health services. AI-powered medical applications—such as triage chatbots, automatic thermal screening cameras, ultrasound diagnostic devices, and post-surgery image analysis apps—use algorithms to construct knowledge from large datasets and make medical decisions based on the processing of the patient’s personal data or profile. This automation of medical decision-making could enhance the quality and efficiency of healthcare services in the European Union (EU),^1^ but at the same time raises concerns for the protection of human rights, and individual patients’ rights in particular.

One problem is that current national health laws in the EU Member States are not necessarily adapted to algorithmic developments,^2^ since they have not made their architecture of patients’ rights fit for the digital age. In fact, although being rooted in the EU and international human rights framework, individual patients’ rights are mainly regulated at the level of the EU Member States. With some intra-national variations, all Member States protect the core patients’ right to health privacy, encompassing the rights to (i) respect for patients’ autonomy; (ii) medical data protection; and (iii) physical integrity. If the regulatory framework is not updated, these rights are threatened by the implementation of automated decision-making in healthcare.^3^

In the context of medical ethics, some have argued that a *patient’s right not to be subject to automated medical decision-making* would be beneficial for the protection of patients.^4^ However, considered from a legal perspective, such a right is not part of the traditional portfolio of patients’ rights, and legal scholars have not yet addressed the question how such a right could be implemented. Current national health laws in the EU Member States do not directly equip patients with the legal means to *refuse* medical procedures based on decisions taken with the aid of assisting AI (eg diagnostics or treatment selection) and medical procedures that make use of partially and fully automated decision-making (eg AI cardiac monitoring or precision medicines).^5^ The EU’s AI strategy could have offered a suitable platform to introduce this right, but this was not the case. Indeed, the EU AI Act only provides for one individual right for persons affected by AI applications, namely in Article 86: the right to explanation of individual decision-making. However, this right explicitly excludes explanations of decisions made with the use of AI medical devices.^6^ Similarly, the upcoming European Health Data Space (EHDS) Regulation does confer individual rights upon patients to control how their electronic health data are used by healthcare providers, but it does not provide for a right to generally *refuse* automated medical decision-making.^7^

In the absence of a direct reference to a *patient’s right to not be subject to automated medical decision-making* in the law, the GDPR may provide a possible pathway to protect the same interests that such a right would safeguard. Indeed, Article 22 GDPR provides individuals with the right ‘not to be subject to a decision based solely on automated processing, including profiling, which produces legal effects concerning him or her or similarly significantly affects him or her’. Deploying the GDPR has two key advantages. First, it is often easier for patients to invoke than some patients’ rights protections enshrined elsewhere in the legal system because of its established enforcement mechanisms and harmonized legal nature, and secondly, the GDPR requires to provide the patient with adequate (organizational and/or technical) tools to meaningfully exercise their rights, preferably built into the system.

Whether Article 22 could be added to the architecture of patients’ rights in order to protect the *right to not be subject to automated medical decision-making* is however moot. The interpretation of this provision has been extensively debated in legal scholarship, mainly in relation to the scope of application,^8^ the (in)effectiveness of rights and safeguards provided in the GDPR in connection to Article 22,^9^ and unclarity about the existence of a ‘right to explanation’ of automated decisions in the GDPR.^10^ However, in December 2023, the Court of Justice of the EU (CJEU) first provided an interpretation of Article 22 in C-634/21 (SCHUFA) in the context of credit scoring.^11^ This case provides insights into interpreting this legal provision in practice. Indeed, it may also clarify the application of Article 22 to medical decision-making.

By examining whether Article 22 GDPR could add an extra layer of health privacy protection if invoked as an *individual patient’s right* in the context of automated medical decision-making, this article makes two key contributions to the existing literature: (i) it problematizes automated decision-making in healthcare from an EU patients’ rights perspective and (ii) it provides a critical analysis of the application of Article 22 GDPR to the medical context in light of the recent SCHUFA judgment, offering new insights into the practical effectiveness of this heavily debated provision. While this article focuses on the EU context, its considerations are useful outside of the EU, as generally patients’ rights are derived from similar international human rights and medical–ethical standards.

This article proceeds as follows. Section II provides an overview of recent developments in automated medical decision-making and highlights its potential threats to patients’ rights—especially the right to health privacy. Section III explains that some of these threats could be mitigated by having a patient’s right to not be subject to automated medical decision-making, which is—however—currently missing. Sections IV and V conduct a legal case study on the application of Article 22 GDPR to automated medical decision-making following the SCHUFA ruling and the contribution of its accompanying safeguards and rights to patients’ rights protection. Section VI proposes the outlines of a health-conformant reading of Article 22 GDPR and draws conclusions.

## II. AUTOMATED MEDICAL DECISION-MAKING: NEW THREATS TO PATIENTS’ RIGHTS

AI technologies in healthcare have the capability to construct knowledge from large datasets, which can be deployed for both virtual (ie diagnosis software) and physical (ie robot surgeons) applications.^12^ Automated decision-making in the healthcare sector differs from automated decision-making in other sectors (ie credit scoring) because these decisions can directly impact the body, health, and life of the patient involved. Experts expect the level of automation in medical decision-making to gradually increase in the next years, which brings about new risks for the protection of individual patients’ rights.^13^ Patients’ rights are a subset of human rights specific to the context of healthcare centred around the patient–health professional relationship, derived from the notion of human dignity and rooted in the EU and international human rights framework and medical–ethical principles.^14^ Patients’ rights deserve specific protection because of patients’ position of vulnerability and dependency when in need of healthcare.^15^*The right to health privacy* is a core patients’ right and compromises several entitlements, rights, and obligations. However, at the moment, a *specific* right for patients not to be subject to automated medical decision-making cannot be derived from the traditional framework portfolio of patients’ rights.^16^

This section highlights the threats of medical automated decision-making to patients’ rights. It first describes the outlines of the right to health privacy. Subsequently, it presents real-world examples of AI tools of different automation level and their application. Finally, it illustrates the risks AI tools present in relation to health privacy.

### A. Components of the patients’ right to health privacy

Privacy scholars generally distinguish between different dimensions of privacy, most commonly *informational (the protection of personal data)*, *decisional (the protection from heteronomous influence in individual decisions)*, and *locational* privacy (the protection of the physical living space).^17^ In the health context, all three dimensions of privacy come into play, and they significantly impact the conceptualization and outreach of some key patients’ rights that are protected in all EU Member States. These collectively characterize what can be considered as a right to *health* privacy and consist of: (i) respect for patients’ autonomy; (ii) medical data protection; and (iii) physical integrity.^18^ These rights are safeguarded at various levels in the legal order applicable to many European states (ie national laws and policies, EU fundamental rights law, and Council of Europe instruments), and a specific framework for protection of personal data is provided for in the GDPR. Despite regulating the processing of data (and protection therefrom) in all sectors, the GDPR is also particularly relevant for the medical context and as a legal instrument contributing to the safeguarding of health privacy. This set of rights—whose implementation is fundamental for the protection of health privacy—is however seriously challenged by the increasing use of automated medical decision-making.^19^

### B. Different levels of automation: assisting AI, partial automation, and full automation

The most basic AI tools are *assisting AI systems* (sometimes referred to as AI clinical decision support systems). These can aid health professionals to make a medical decision about an individual patient by providing suggestions. In general, such AI systems automatically process personal data to come to a medical decision, and the health professional can choose whether to take over the suggestion in their provision of patient care. An example of an assisting AI system is an image-based AI tool for skin cancer diagnosis.^20^ The application classifies an image of an individual patient’s skin lesion as benign or malignant. The idea is that health professionals can look both at the original image and at the classification made by the tool, to then make a diagnostic decision about an individual patient.^21^ Similar AI tools exist for treatment recommendations, where the system processes individual patient data (eg electronic health records and self-reported systems) to evaluate the prognosis of certain treatments for the specific patient, such as AI breast cancer therapy selection.^22^

Stepping up one level in terms of automation, there are *Partially automated medical decisions systems.* These consist of AI systems that take the medical decision, but ask for human input in certain instances. A first example is AI semi-automated diagnosis: the system classifies images in diagnostic categories (positive/negative), and the original image is only presented to the health professional in borderline cases.^23^ Another example is AI for clinical trial selection. By scanning through a large database of patient data (eg electronic health records and medical images), the process of identifying patients who are eligible for a specific clinical trial is automated.^24^ The actual selection still depends on a human decision. A third example is AI monitoring of cardiac patients. This tool automatically analyses personalized heart rate data collected by a wearable or implantable device. It detects arrhythmias and automatically transmits the relevant information to the patient’s cardiologist.^25^

Finally, *Fully automated medical decisions systems* are those AI tools where the system alone makes choices without—in principle—any human involvement. While full automation is still not entirely possible—it could, for example, be developed for AI insulin systems.^26^ In non-AI automated insulin systems, patients need to provide the system with personal data about food intake and exercise, in order to calculate the level of insulin the wearable insulin pump automatically delivers.^27^ In AI insulin systems, sensor data are combined with other data sources, such as activity data from a fitness tracker, geolocation on the smartphone, and hand-gesture sensing. Over time, it can recognize certain patterns in the individual behaviour and automatically deliver insulin accordingly. Another example is autonomous surgical robots, where an AI system can locate a tumour through image analysis and sensors, then decide on the best location to make an incision in the body, and sometimes autonomously perform the surgery.^28^ A third example is AI precision medicine in oncology, where AI is used to detect patterns in large datasets in order to identify a specific patient’s molecular profile to match with a specific cancer medicine.^29^

### C. Divergent risks for patients: from errors, to access, to autonomy

Regardless of the level of automation, a general threat that the use of AI systems poses to health privacy concerns the fact that AI development (and application) depends on high-quality data. However, high-quality health data are difficult to obtain, as it is often inaccurate (errors in medical records) and/or biased (lack of inclusive clinical data).^30^ This can lead to errors in the AI systems, and thus also in the medical decision-making they contribute to, potentially resulting in physical harm and threatening physical integrity—one interest safeguarded by health privacy. Another issue is that AI is prone to biases that can lead to discriminatory health outcomes.^31^ AI tools for skin cancer diagnosis may, for instance, perform better for White people than Black people because Black people were underrepresented in the training dataset.^32^ In general, marginalized groups are more prone to the health risks of automated medical decision-making, challenging their autonomous decision-making powers.^33^ Automated medical decision-making can create new barriers to access to healthcare. For example, for AI cardiac monitoring, patients are required to have a wearable or smartphone. Digital divide factors such as low levels of digital literacy or access to technology impact overall access to healthcare—preventing patients from autonomously deciding on the care they need.^34^ Some AI tools can also bring about trust issues because of their common lack of transparency, for example, in the case of autonomous surgical robots. The difficulty in establishing patients’ trust and acceptance may deter some patients from seeking healthcare.^35^ Along the same lines, automated decision-making in health challenges human dignity. Increasing use of AI may depersonalize interactions in patient care and neglect individual human characteristics.^36^ In general, empathy and empathic communication are important factors in healthcare, and as AI is (still) incapable of empathy, automated decision-making risks reducing humans to numbers—impacting the core values of patients’ rights.^37^

As AI systems collect, share, and combine large amounts of personal data—often sensitive health data—they introduce new risks to the privacy of patients. New risks for disclosures of personal data are first caused by the increased involvement of commercial third parties—such as tech developers and data storage companies—in the realm of healthcare. This pushes principles such as purpose limitation to their boundaries, thus threatening individual self-determination. Moreover, because of the need for enormous amounts of personal data to create AI systems, AI developers are incentivized to push legal and ethical boundaries to maximize personal data collection. The ‘blending’ of different sources of personal data—for example, in the development of AI insulin systems—leads to the creation of an elaborate ‘health profile’ of the patient, which contains sensitive details about their personal life and health status. This information can also be used to influence or manipulate personal decisions, such as purchasing decisions.^38^ If the data security of the AI tools is not guaranteed, for example, with AI cardiac monitoring, confidential personal health data can be revealed and used for the wrong purposes, such as commercial targeting or law enforcement. If personal data are processed by and transferred to multiple parties, the right of patients to data protection is challenged, as it becomes difficult to exercise meaningful control over their personal data.

Additionally, there is usually a lack of explainability in medical AI systems: systems are ‘black boxes’ and do not always allow for identification and adequate understanding of the relevant parameters of the system and their significance for a certain decision.^39^ This is often inherent to the specific system, for example, because the choice was made to prioritize effectiveness over interpretability, which is frequently the case in the field of healthcare.^40^ Current post-hoc explainability methods, such as saliency maps, do not necessarily provide the information needed for human understanding.^41^ The lack of explainability makes it difficult for both health professionals and patients to understand how the system reached a certain medical conclusion. This is, for example, problematic in the context of AI precision medicine, where often the final decision of the AI system is based on thousands of variables. This may impair patient autonomy, as the information they would need to make an informed decision would not always be available.^42^ In this respect, it may also become difficult for patients to provide valid informed consent to automated medical decision-making, as (i) health professionals may not be required to disclose the use of AI in every step of the medical decision-making process and (ii) alternative, non-AI treatment may not always be available.^43^ When the AI decision has direct effects on the patient’s body, such as with AI insulin systems, this may also affect the patient’s physical integrity.

Considering these risks, bioethics scholars suggested that a *right not to be subject to automated medical decision-making* can help to avoid health privacy being considerably compromised. ^44^ Such a right entails that—under certain circumstances—patients should have the right to refuse medical procedures based on decisions taken with the aid of assisting AI (eg diagnostics or treatment selection) and from the use of partially and fully automated decision-making (eg AI cardiac monitoring or precision medicines) as part of their individual medical treatment.^45^ However, as explained in the next section, such a right is currently absent in the EU patients’ rights framework.

## III. LACK OF A PATIENT’S RIGHT NOT TO BE SUBJECT TO AUTOMATED *MEDICAL* DECISION-MAKING

The right to health privacy as implemented in current law does not necessarily encompass a right *not to be subject to automated medical decision-making*. Indeed, at both the EU and the Council of Europe levels, no such right is explicitly recognized. Moreover, also national interpretations of core patients’ rights and related policies do not explicitly specify the rights of patients in relation to automated medical decision-making. For example, it is unclear whether it can be derived from the right to adequate information that health professionals are required to disclose the use of AI in every step of the medical decision-making process. If no such duty exists, this can cause problems for health privacy, since not disclosing to patients that AI was used in the decision-making process has direct consequences for the right to self-determination, as patients cannot approve or refuse the use of an AI system if they are not aware of its use.^46^

Whilst not present explicitly in the European regulatory framework, it seems also difficult to implicitly derive a right not to be subject to automated medical decision-making from other legally relevant sources composing the patients’ right framework. For example, it can hardly be derived from medical confidentiality obligations. These do not protect the patient from being subject to automated medical decision-making, as they allow health professionals to discuss patient information with other health professionals in the treatment team without informing the patient. The same exception may apply when the patient’s information is shared and processed by the assisting AI tool.^47^ The general right to physical integrity may also be a potential candidate to derive a right not to be subject to automated medical decision-making. Indeed, it does enable patients to refuse to be subjected to automated medical decisions, such as autonomous robot surgeries or partially automated diagnostics. However, it does not encompass *a right to human intervention* nor does it guarantee patients *access to alternative, non-AI treatment*. If no alternative non-AI treatment is available, this impacts the patient’s right to access healthcare, rendering the right useless in practice.^48^ In sum: it seems that at the moment, a *specific* right not to be subject to automated medical decision-making is not part of explicit European regulation, nor can it be derived from the traditional framework portfolio of patients’ rights.^49^

However, while not specifically addressing the medical decisions, Article 22 GDPR provides individuals with the right ‘not to be subject to a decision based solely on automated processing, including profiling, which produces legal effects concerning him or her or similarly significantly affects him or her’. In this sense, Article 22 GDPR puts forward a *general right* not to be subject to automated decision-making. In theory, a nuanced interpretation of this provision may provide the missing puzzle piece for the protection of patients against the detrimental effects of AI in healthcare, and indirectly grants a similar level of protection for health privacy as an *explicit* right not to be subject to automated medical decision making would.

While it would be possible to explore other legal pathways to enforce this right,^50^ the nature of the GDPR offers some procedural benefits. First, in some cases, the GDPR is easier for patients to invoke than some patients’ rights protections enshrined elsewhere in the legal system, because of the existence of both independent national data protection authorities and data protection officers in healthcare institutions. It is, however, important to note that the GDPR was not intended as a health law instrument nor is it focused on the protection of the rights of patients as such. Indeed, if evaluated from a patients’ rights perspective, the main challenge of the GDPR seems to be its interplay with national patients’ rights, health data rules, and medical ethics. All EU Member States have long had their own laws and policies on health data protection in place based on the principle of medical confidentiality.^51^ At the same time, the harmonized nature of the GDPR may be of added value to smoothen the ‘patchwork’ of patients’ rights in the Member States, often consisting of legal instruments, ethical codes, and professional protocols.

Secondly, Article 22 GDPR introduces individual rights that could be invoked by patients subjected to medical automated decision-making. The most useful effect of the individual rights introduced in Article 22 GDPR for patients seems to be the requirement to provide the patient with adequate (organizational and/or technical) tools to meaningfully exercise their rights as part of the decision-making process. The situating of this right within the GDPR—which also promotes the accessible exercise of rights, preferably built into the system (‘privacy-by-design’), supports the implementation of rights within the system itself: in some way, a ‘rights-by-design’. For instance, in the case of AI insulin systems, the system could record the exact grounds on which a certain decision was based (ie food intake or activity), connected to a system where the patient could request further information about the decision. In this way, an additional layer of protection for patients could be created: on top of the patients’ rights flowing from the relationship with the health professional, patients may be equipped with rights towards the AI tool itself. This could take away potential burdens to exercise rights, particularly in case of lengthy legal procedures. Finally, the default prohibition of automated decision-making in Article 22 GDPR may prevent particularly harmful decision-making practices in the medical context, for example, an automated decision to refuse a patient access to emergency care based on their medical history.

However, the interpretation of Article 22 GDPR has been a topic of debate in legal scholarship. The next section provides a critical analysis of the application of Article 22 GDPR to the medical context, using the recent ruling of the CJEU on its interpretation.^52^ While this case concerned the context of credit scoring, it may also clarify the application to medical decision-making.

## IV. POST-SCHUFA: THE RIGHT NOT TO BE SUBJECT TO AUTOMATED *MEDICAL* DECISION-MAKING IN THE GDPR

Article 22 of the GDPR protects the right not to be subject to decision-making based solely on the automated processing of personal data. The predecessor of the GDPR, the Data Protection Directive already contained a right similar to the GDPR’s Article 22, namely a right not to be subject to a decision based on the automated processing of personal data intended to evaluate certain personal aspects relating to the data subject.^53^ This right was accompanied by an access right to knowledge of the logic involved in any automatic processing of data concerning him.^54^ By adding this provision to the directive, the European Commission aimed to safeguard individual people’s capacity to influence decision-making processes that affect them,^55^ and prevent human decision-makers from escaping responsibility by shifting it to machines.^56^ Another reason for adoption was the prevention of objectification of individuals and the protection of human dignity.^57^ Under the GDPR, Article 22 was introduced for similar reasons—although slightly broadened—specifically because of concerns about possible technical deficits and unfair discrimination.^58^ Authors have argued that the right provided by this article is based on the three pillars of transparency, contestability, and accountability.^59^ But to what extent is Article 22 GDPR applicable in the medical context? The recent SCHUFA ruling may clarify its scope of applicability.^60^

### A. A brief introduction to automated decision-making in Article 22

Article 22 GDPR states that ‘*The data subject shall have the right not to be subject to a decision based solely on automated processing, including profiling, which produces legal effects concerning him or her or similarly significantly affects him or her.*’

Paragraph 2 of Article 22 contains three exemptions to this right:

1. if the decision is ‘necessary for entering into, or performance of, a contract between the data subject and a data controller’;
2. if the decision is ‘authorised by Union or Member State law to which the controller is subject and which also lays down suitable measures to safeguard the data subject’s rights and freedoms and legitimate interests’; and
3. if the decision ‘is based on the data subject’s explicit consent’.

Paragraph 3 of Article 22 stipulates that—in case of exemptions (1) and (3)the data controller must adopt suitable measures to protect the data subject. Minimum safeguards are (i) the right to obtain human intervention on the part of the controller, (ii) the right to express his or her point of view, and (iii) the right to contest the decision. Recital 71 adds the following safeguards: (iv) to provide specific information to the data subject and (v) the right to obtain an explanation of the decision. Paragraph 4 of Article 22 prohibits decision-making based on special categories of personal data as protected under Article 9(1) GDPR, ‘unless point (a) or (g) of Article 9(2) applies and suitable measures to safeguard the data subject’s rights and freedoms and legitimate interests are in place.’ Thus, automated decision-making can be based on the processing of personal data if the decision is based on explicit or if processing is necessary to protect public interest, and suitable protective measures are in place.

Article 22 is accompanied by other transparency requirements in the GDPR. Data controllers must always be able to demonstrate that personal data are processed in a transparent manner in relation to the data subject.^61^ Articles 13 and 14 GDPR introduce general information obligations for data processing to guarantee transparency.^62^ Data controllers have specific transparency obligations when it comes to automated decision-making: information obligations under Articles 13(2)(f) and 14 (2)(g) GDPR and a data access right under Article 15(1)(h) GDPR. Data subjects should be informed about the existence of automated decision-making and receive meaningful information about the logic involved, as well as the significance and the envisaged consequences of such processing for the data subject.^63^ According to the European Data Protection Board (EDPB), the data subject must be given generic information that is also helpful for him or her to contest the decision, specifically on the deliberations in the decision-making process, and on their respective weight on a general level.^64^

### B. Applicability of Article 22 GDPR in the medical context

The applicability of Article 22 GDPR depends on three cumulative conditions: (i) there must be a ‘decision’; (ii) that decision must be ‘based solely on automated processing, including profiling’, and (iii) it must produce ‘legal effects concerning [the interested party]’ or ‘similarly significantly [affect] him or her’.^65^ Recently, the CJEU explicated these conditions in a preliminary ruling on the situation in which a private company (SCHUFA) provided its clients with information on the creditworthiness of certain individuals (eg a prognosis on whether a person will repay a loan), by calculating a probability value or ‘credit score’ on the basis of certain characteristics of the individual. Clients use these credit scores to decide whether to grant a loan to the individual applicant.^66^

In the SCHUFA case, the CJEU has confirmed that the concept of ‘decision’ has a broad scope and also includes ‘measures’ or ‘acts’, such as the automatic refusal of a request without human intervention (eg an online credit application).^67^ A probability value that predicts an individual’s behaviour (eg in relation to creditworthiness) can also be seen as a ‘decision’ in this sense.^68^ All AI tools used for medical decision-making make use of automated processing of personal data and will (at some point, and depending on the level of AI automation) result in a medical decision regarding an individual patient. In the case of full automation, it can be argued that the AI’s outcome is equivalent to the ‘decision’, similar to the automatic refusal of an online credit application. The CJEU’s rejection of the narrow interpretation of what constitutes a ‘decision’, also opens the door to medical AI, with lower levels of automation, for example, AI systems advising on the eligibility of a patient to participate in a clinical trial. This advice can also be seen as a ‘decision’, even though the health professional makes the final decision on the selection of clinical trial participants. In this light, the A-G has argued that if there is a significant and decisive influence of the AI’s output on the final decision regarding the individual, ‘the fact that a third party takes the final decision’ does not change that this decision is ‘based on automated processing’. The A-G adds that a narrow interpretation would undermine the objective of the GDPR to protect individuals against automation with transparency rights, as these only apply to ‘decisions based on automated processing’.^69^

The second condition is that the decision ‘is based solely on automated processing, including profiling’. The word ‘solely’ indicates a very limited scope of application, whereby any human involvement in the decision-making process nullifies the prohibition. However, the EDPB has interpreted the scope of Article 22 more broadly by explaining that human involvement must be ‘meaningful’.^70^ The involvement must be performed by a competent person who is also competent to change the decision.^71^ In the case of full automation, it can be argued that there is no meaningful human involvement in the decision-making process. However, when the decision is only partially automated or assisting, such as the use of AI tools for the diagnosis of skin cancer, it is doubtful whether this would qualify as *solely* automated decision-making in the sense of Article 22 GDPR because of uncertainties about the actual weight the health professional assigns to the AI’s diagnosis. On the one hand, it could be argued that it is in fact the health professional that makes the central decision that has effects on the patient. The diagnosis provided by the AI tool is just advice and the health professional can decide not to follow it. On the other hand, there is increasing evidence that health professionals are likely to act upon the decision of an AI device because of ‘automation bias’ or ‘overtrusting technology’^72^: trusting the AI’s diagnosis of a specific patient’s skin lesion more than their own.^73^ In this light, it is questionable whether one could consider the health professional’s involvement meaningful. Here, the SCHUFA judgment does not necessarily provide any new insights, since the Court rules that there is no doubt that the situation at hand (‘the automated establishment of a probability value based on personal data relating to a person and concerning that person’s ability to repay a loan in the future’) meets the definition of ‘profiling’ in the GDPR.^74^ However, given the broad definition assigned to ‘decision’ by the Court, a similar interpretation of this criterium is not unthinkable.

Finally, the decision must produce ‘legal effects concerning [the interested party]’ or ‘similarly significantly [affect] him or her’. While the decision itself does not constitute any *legal effects*, many examples of automated medical decisions will *significantly affect* the patients, since AI tools either have direct effects on the body (eg AI insulin systems or autonomous surgery robots) or make decisions that indirectly affect the health status of the patients. For example, it is fair to assume that a skin cancer diagnosis or breast cancer treatment selection has a significant, prolonged, or permanent impact on the patient involved.^75^ Both a correct diagnosis of the skin lesion as benign or malignant, and a diagnostic error, have significant effects on the patient’s life, as further important medical treatment decisions are based on this. However, whether these effects are realized depends—again—on how much weight the health professional assigns to the AI’s diagnosis. In the SCHUFA case, the Court explained that the probability value (the ‘decision’) has significant effects on the consumer applying for a loan because empirical research shows that ‘an insufficient probability value leads, in almost all cases, to the refusal of that bank to grant the loan applied for’.^76^ For fully automated medical decisions, the same argument will apply. However, generally, for assisting AI tools, the impact on the final medical decision is less evident. While empirical research gives us reason to believe that AI technology has stronger effects on human behaviour than non-AI technology,^77^ healthcare professionals do not necessarily ‘blindly follow’ the AI’s advice.

Thus, the applicability of Article 22 GDPR depends on the following unresolved question: when making use of AI tools for medical decision-making, what is the meaning of the health professional’s involvement in the decision-making process? This probably needs to be determined on a case-to-case basis. At the same time, the SCHUFA ruling seems to open the door to a broad interpretation of the scope of application of Article 22 GDPR—in favour of individuals.

## V. RIGHTS AND SAFEGUARDS AGAINST AUTOMATED *MEDICAL* DECISION-MAKING IN THE GDPR

It follows from the above that the scope of application of Article 22 for automated medical decisions is still uncertain. If applicable, however, the corollaries of this article and other rules in the GDPR provide for more rights and safeguards against automated decision-making. Article 22(3) GDPR provides individuals with several minimum rights when subjected to automated decision-making: the right to human intervention, expressing points of view, contesting the decision, and explanation of the decision. Article 22(4) GDPR stipulates that automated decision-making based on *health* data is only allowed under specific conditions. On top of the condition of a valid legal ground for the processing of the health data to be used in the automated decision, the decision-making needs to be either (i) strictly necessary for contractual purposes, (ii) authorized by law by Member States or the EU, or (iii) explicitly consented to.^78^ This section evaluates these rights and safeguards from a patients’ rights perspective and health privacy perspective.

### A. The safeguard of ‘explicit consent’ for patients

Article 22(2) GDPR proposes explicit consent to automated decision-making as a safeguard. In the GDPR, consent means the freely given, specific, informed, and unambiguous indication of the data subject’s agreement, expressed by a statement or a clear affirmative action.^79^ Consent requires ‘real choice’ and should be ‘granular’ and ‘specific’. The term ‘explicit’ implies that the data subject must give an ‘*express* statement of consent’: the ‘ticking of boxes’ is not sufficient.^80^

There is, however, a discrepancy between the rights to informed consent as understood in health law, and informed consent in data protection law.^81^ The health professional has the ethical and legal responsibility to enable a specific patient to make an informed decision about medical treatment by exchanging information about the benefits and risks of the course of treatment, potential alternatives, and consequences of the patient’s decision. The patient’s right to information is not absolute but requires the health professional to strike a balance between under-informing and information overload, tailored to the specific patient.^82^ In this line of thought, informed consent to medical decisions is vital for the protection of patient autonomy, self-determination, and physical integrity.^83^ In data protection law, on the contrary, consent does not serve as a general safeguard or right but as a legal basis for the processing of personal data, among other legal bases. The GDPR, for example, states in Articles 6 and 9 that consent is amongst multiple potential legal bases whereupon health data can be processed, but an alternative legal basis for data processing can be found in the existence of a relevant public interest, such as collecting data about infectious diseases, or for scientific purposes.^84^

However, in the privacy debate, informed consent to processing of personal data is considered the main solution to empower data subjects.^85^ This also seems to be the rationale behind the GDPR’s regime for sensitive personal data, where the threshold is raised to ‘explicit’ consent, apparently to add an extra layer of protection. Data protection scholars have, however, long expressed fundamental concerns about how an individual’s (explicit) consent can lead to better protection of (medical) data protection. While the GDPR prescribes the correct requirements for obtaining valid consent, these requirements seem impossible to meet in practice.^86^ Hence, first, there is a de facto *lack of freedom* to give consent in practice, because of power imbalances between patients and health professionals.^87^ Furthermore, with respect to health data processing, patients often have no choice if they desire adequate medical treatment. While informed consent in health law requires access to alternative treatment, this is not part of the GDPR.^88^ Secondly, there is a *lack of real information* for patients giving consent in practice, as there is an inherent risk of information overload, lack of ability to truly understand, and consent desensitization.^89^ For example, in the case of AI tools for precision medicine, the complexity of the tool makes it very difficult for patients to provide valid informed consent. Because of this, in many cases, consent on the processing of data is a mere ‘ticking the boxes’ exercise, and it can thus be doubted that it provides adequate safeguards for health privacy in respect to AI.

### B. A patients’ right to human intervention?

Any discussion around the rights and safeguards with respect to the use of AI in medical decision-making also begs a diametrically opposite question: Is there a right to be treated by a human health professional? When health data are processed for automated decision-making, it follows from Article 22(3) GDPR that the involved individual has the right to obtain some form of human intervention. This intervention should likely happen in the final stage of the decision-making process that can either confirm or change the automated decision—as involving a human decision-maker in an earlier stage would render Article 22 inapplicable, since it would turn the decision into one that is not based solely on automation. Human oversight is often advocated as a central ethical value for AI deployment.^90^ The rationale of this is that human oversight can function as a safeguard to help ensure that an AI system does not undermine patient autonomy or cause untransparent decision-making, privacy and data protection issues, or discrimination.^91^

In theory, equipping patients with the right to human intervention in automated medical decision-making could contribute to patients’ rights and health privacy specifically (especially in relation to self-determination and physical integrity) in several ways. First, including a health professional in the automated decision-making process could soften the negative effects of the ‘objectivation’ of patients or reduction of patients to numbers, bringing back the core condition of human dignity, and bringing moral values into the automated process. This could also contribute to the establishment or maintenance of trust in the patient–health professional relationship, which is an essential prerequisite for patients’ access to healthcare. Research also shows how human involvement in medical decision-making—as opposed to full automation—is crucial for empathy and compassion, values that directly impact health outcomes.^92^ Secondly, in theory, health professionals could use their medical knowledge and expertise to test the accuracy of the automated decision for a specific patient, which may mitigate the risks of physical harm and allow patients to make more autonomous decisions about their bodies and health. For example, when AI tools are used for diagnostics, health professionals could fulfil the role of controller of potential biases in the outcome of the decision (eg to account for different symptoms for cardiac arrest in men and women), strengthening the patient’s right to physical integrity. Including a health professional could potentially also strengthen the right to adequate information, informational self-determination, and medical data protection, as the health professional is—in addition to the provisions in the GDPR—bound to (i) medical confidentiality and (ii) medical informed consent duties.

However, in practice, it is questionable how exactly meaningful human oversight can be implemented in automated medical decision-making. First, it is doubtful whether the health professional can fulfil a meaningful role in the decision-making process because of the complexity and opacity of many automated decision-making systems. Sarra argues that, as intelligent systems are deployed to make decisions ‘because of their inhuman efficiency’, it is very difficult for the human involved to understand what went wrong in a specific decision and justify the need to change the automated decision.^93^ Moreover, a recent empirical study by Jabbour and others shows that it is very difficult for clinicians to recognize systematically biased AI models, even when image-based AI model explanations are provided.^94^ In this light, the involvement of a health professional in the final stage of the decision-making process will offer little protection against AI-powered decisions causing (physical or mental) harm and may even legitimize them.^95^

Secondly, research from social psychology suggests that humans often over-rely on automated systems. There is an ‘automation bias’: the tendency to follow computer-generated outcomes over human-generated ones. For example, a study on oncologists classifying mammograms as either ‘further examination required’ or ‘no further examination required’ with the aid of computer systems advising on the classification showed the influence of the computer’s decision on the oncologists’ behaviour. A significant number of oncologists (i) neglected to take appropriate action when the computer failed to detect the irregularity in the mammogram because of decreasing human vigilance (errors of omission) and (ii) for ambiguous mammographs, using the computer’s absence of prompts as a reassurance not to invite the patient for further examination.^96^ A recent study on automation bias in inexperienced, moderately experienced, and very experienced radiologists when reading mammograms with the aid of AI systems showed that all radiologists are prone to automation bias when being supported by an AI-based system, irrespective of experience level.^97^ This over-reliance on AI advice is conceptualized by Strauß as ‘deep automation bias’.^98^ Another concern is the occurrence of ‘selective adherence to algorithmic advice’: human decision-makers tend to rely on automated decisions selectively: when their predictions correspond to stereotypes.^99^ It is questionable to what extent the phenomenon of automation bias decreases the value of human intervention as a safeguard for patients. It has been argued, for example, that adequate education and training on the use and limitations of AI systems can minimize the occurrence of automation bias.^100^ Moreover, as argued by Kostick-Quenet and Gerke, additional user testing of medical AI tools in settings that resemble the intended use can also minimize the risks of ‘blind’ overreliance on AI by addressing context-related risks at an earlier stage, for example, by providing detailed use instructions.^101^ Especially for AI tools with lower levels of automation, such as diagnostic tools, human oversight by health professionals with adequate AI skills can function as a safeguard for patients.

However, along the same lines, increasing automation poses risks to the quality of the health professional’s medical skills and knowledge levels. When health professionals over-rely on the capacities of AI tools, they may lose the necessary expertise to intervene in an automated medical decision.^102^ If ‘deskilling’ of health professionals is a real risk, their involvement in medical decision-making would not be meaningful, and thus patients would not necessarily benefit from a right to human intervention with regard to the protection of their safety. Potentially, overreliance on AI tools could also lead to ‘loss of self-confidence and affect the willingness of a physician to provide a definitive interpretation or diagnosis’.^103^ Another issue could be that it is questionable whether health professionals would feel the freedom to challenge the automated decision, because of uncertainties about attribution of responsibility, accountability, and liability, and fear of lawsuits.^104^ For example, in the case of fully automated surgery, are human surgeons capable of stepping in and replacing the robot if something goes wrong? The risk of ‘deskilling’ could complicate human intervention in general, but for automation in ‘microsurgery’, such as tumour removal with very small equipment, surgery ‘by hand’ could be impossible because of the high degree of required precision.^105^

On top of this, the GDPR only provides a right to human intervention in the final decision. This causes two issues for the protection of patients. First, the harm may have already taken place, such as physical harm caused by autonomous robot surgery or the AI insulin system, or the health effects of a delayed diagnosis.^106^ Intervention in an earlier stage of the decision-making process, where there are still significant other pathways to consider, would be of more use to patients. In this sense, the right does not necessarily strengthen patient autonomy. Secondly, the GDPR does not give patients a right to human decision-making *instead of* automated decision-making. The human may be included in the automated decision at some point, but at this stage, some harms may have already occurred and are not easy to turn back, for example, the processing of personal data. In order words, the automated processing has already taken place, with potentially detrimental consequences for the protection of medical data protection and informational self-determination. To illustrate, the patient could request human intervention over an AI recommendation for a specific type of cancer treatment, but their personal medical data would already have been processed to generate the decision.

### C. A patient’s right to expressing points of view and to contest the decision?

Article 22(3) GDPR puts forward a right for data subjects to express their point of view. The data controller must implement suitable measures to safeguard this right. The right to expressing points of view seems to relate to expressing views in the case of (i) asking for human intervention or (ii) contesting the decision.^107^ While in theory, the sharing of views, opinions, and preferences does strengthen patient autonomy as it enhances self-determination and physical integrity, Article 22(3) does not stipulate an obligation for either the AI tool or the involved human to act upon this expression. In that sense, it does not seem to provide any direct extra protection to a patient subjected to automated medical decision-making. On the other hand, the fact that measures must be implemented in the decision-making process in order for patients to exercise this right may, in practice, lead to the (voluntary) consideration of patients’ opinions.

In addition, data subjects are granted the right to contest the decision resulting from the automated decision-making process. To this end, there must be suitable measures to ensure that patients have access to this right. The right to contest the decision is different from the right to human intervention, as requesting human intervention does not equal a request to change the outcome of the automated decision-making process. Similarly, exercising the right to contest the decision does not seem to require the involvement of a human—disputes may also be settled in an automated manner.^108^ In any way, the GDPR requires the implementation of a ‘contestable system’, equipping patients with the practical tools to contest the automated decision.^109^ The implementation of such a right *within* the system could add an extra layer of protection for patients, in addition to, for example, the patient’s right to refuse a specific treatment.^110^ However, a key concern about the effects of a right to contest the decision is the patient’s lack of information about the decision-making process: it is very difficult to contest a decision without fully understanding how it was taken by the machine. This concern is often linked to ‘the right to explanation’.^111^

### D. A patient’s right to explanation?

The nature of a potential ‘right to explanation’ of automated decisions in the GDPR has been a topic of extensive scholarly debate. Articles 13 and 14 entail specific transparency obligations when it comes to automated decision-making and require data controllers to inform the data subject about the following: (i) the fact that they are engaging in this type of activity; (ii) provide meaningful information about the logic involved; and (iii) provide information on the significance and envisaged consequences of the processing. The CJEU explains that transparency about personal data processing is important because it is a prerequisite for other rights, such as the right of access to personal data and the right to object to the processing of data.^112^ Brkan adds to this that granting data subjects a right to explanation of automated decisions enables them to ‘understand the reasons behind the decision and to prevent discriminatory or otherwise legally non-compliant decisions.’^113^ While some scholars such as Wachter, Mittelstadt and Floridi accept only a very restrictive interpretation of a right to explanation,^114^ others such as Goodman and Flaxman,^115^ Casey, Farhangi and Vogl^116^ confer from Articles 13 and 14 GDPR’s ‘right to meaningful information about the logic involved’ a solid ‘right to explanation’ of automated decisions for individuals. Selbst and Powles advocate a ‘functional and flexible’ right, which enables individuals to exercise their autonomy and, for example, contest an automated decision.^117^ Furthermore, there is discussion about the *type* of information that must be provided and the *time* of provision (*ex-ante* or *ex-post* the automated decision-making).^118^ According to the EDPB, the data subject must be given information that is also helpful for him or her to contest the decision, specifically on the deliberations in the decision-making process, and on their respective weight on a general level.^119^ Edwards and Veale claim that, even if there was a right to explanation, there would be great difficulty in providing data subjects with meaningful explanations, making it an empty promise in practice.^120^

For the patient involved in automated medical decision-making, information about the decision-making process is a key factor in the protection of their patients’ rights and health privacy. Adequate information is essential to enable patients’ rights related to health privacy, such as the right to refuse treatment. It is also crucial for the protection of the right to medical data protection to provide information about data processing for automated medical decision-making. To illustrate, in the case of AI precision medicine, the patient needs certain information to object to the decision to choose a specific medicine (eg that it was an automated decision, the grounds for deciding on medicine X instead of Y, etc). Active information sharing is also an important aspect of human dignity and is essential for building trust. For example, adequate information about the functioning of an AI insulin system can improve patients’ trust in the system.

However, the lack of judicial clarification on the nature of the GDPR’s ‘right to explanation’ may undermine its effectiveness. The right to informed consent—a long-recognized patient’s right—seems to be a much stronger right, as its core elements have been established by both national courts and the ECtHR, and healthcare institutions have procedures in place to guarantee proper understanding of patients, with the aim of enabling patients’ autonomy and protecting human dignity. For example, the patient’s right to informed consent also requires access to information about alternative treatments, and all medical information must be included in the medical file, which the patient must have access to. In the absence of a uniform interpretation of Articles 13 and 14 GDPR, these provisions will not contribute substantially to the protection of health privacy against automated medical decision-making.

## VII. CONCLUDING REMARKS: THE GDPR AS A CATALYSATOR FOR PATIENT PROTECTION?

Decision-making processes in the healthcare sector are changing quickly, whilst the legal and regulatory framework struggles to keep up. As is often the case in digital transformations, digital processes are evolving faster than the law can adapt. Because of the novelty of these technologies and the fear of being outdated, regulators often favour introducing new legal provisions or instruments over new interpretations of existing legal frameworks, causing the gap between law and technology to grow even bigger. This effect seems to be even stronger in the context of EU regulation, where the balancing of interests in the different EU institutions and political landscape has always been a lengthy process. As EU integration in healthcare is still limited, not much has been said about individual rights in relation to medical technology. The EU’s formal (direct) involvement in medical technology regulation—including medical automated decision-making—does not extend beyond the regulation of the safety and quality of the devices themselves. However, this limitation in EU competency does not prevent general EU legislation—such as the GDPR—and fundamental rights instruments from being applied in the realm of healthcare.

This article examined the impact of AI on health privacy and showed how—in the absence of an *explicit* right not to be subject to automated medical decision-making—other provisions (and in particular Article 22 GDPR) could be used to provide an equivalent level of protection of patients’ rights and health privacy. It showed that many features of Article 22 GDPR can indeed constitute the basis for a satisfying protection of health privacy in respect to developments in medical AI. However, it also showed that the rights and safeguards against automated decision-making provided for in the GDPR do have their limitations when applied in the medical context. At the same time, since a *right not to be subject to automated medical decision-making* is currently missing in other frameworks, the GDPR’s provisions surrounding automated decision-making may still provide patients with an extra layer of protection. Therefore, an adequate level of protection for health privacy could be achieved by a reading of Article 22 GDPR that takes into consideration the specificities of the healthcare context. It is important to note that this health-conformant reading does not imply a blanket prohibition of automated decision-making in the medical context, but rather introduces conditional rights and safeguards.

That said, the practical application of the rights recognized in the GDPR—and Article 22 specifically—remains a key issue. Because of the opacity of most automated decision-making systems, it is not always possible for patients to find out whether a decision was (i) automated and (ii) based on their personal data, making it more difficult to exercise their rights. Furthermore, objecting to the use of automation does not guarantee a different outcome in the decision. Thus, while the GDPR offers a theoretical solution, it may not be as useful in practice.

Simply rebranding the GDPR and its right not to be subject to a decision based solely on automated processing as a safeguard for patients’ rights and health privacy is not sufficient. While the EU data protection law framework introduces a regime of individual legal protection that the current health law framework misses, health-conformant interpretation of the GDPR is necessary. In order for the instrument to be useful in the medical context, we need to interpret it in light of the underlying ethical values that have given way to patients’ rights as protected in the Member States. In this manner, the general rules. of the GDPR can pave the way can pave the way for ultimately developing a special EU-wide patients’ right not to be subject to automated medical decision-making, which will eventually lead to better protection of patients’ health privacy rights.

## Footnotes

1 ‘Artificial Intelligence in Healthcare: Applications, Risks, and Ethical and Societal Impacts’ (European Parliamentary Research Service, STOA 2022).

2 Charlotte Högberg and Stefan Larsson, ‘AI and Patients’ Rights: Transparency and Information Flows as Situated Principles in Public Health Care’ in Katja de Vries and Mattias Dahlberg (eds), *De Lege—Yearbook Uppsala Faculty of Law 2021* (Iustus förlag 2022).

3 Hannah van Kolfschooten, ‘EU Regulation of Artificial Intelligence: Challenges for Patients’ Rights’ (2022) 59 Common Market Law Review 81–112.

4 Thomas Ploug and Søren Holm, ‘The Right to Refuse Diagnostics and Treatment Planning by Artificial Intelligence’ (2020) 23 Medicine, Health Care and Philosophy 107; Thomas Ploug and Søren Holm, ‘The Four Dimensions of Contestable AI Diagnostics—A Patient-Centric Approach to Explainable AI’ (2020) 107 Artificial Intelligence in Medicine 101901.

5 European Commission, ‘Study on eHealth, Interoperability of Health Data and Artificial Intelligence for Health and Care in the European Union. Lot 2: Artificial Intelligence for Health and Care in the EU. Final Study Report’ (Publications Office of the European Union 2021).

6 Regulation of the European Parliament and of the Council laying down harmonized rules on artificial intelligence and amending Regulations (EC) No 300/2008, (EU) No 167/2013, (EU) No 168/2013, (EU) 2018/858, (EU) 2018/1139, and (EU) 2019/2144 and Directives 2014/90/EU, (EU) 2016/797, and (EU) 2020/1828 (Artificial Intelligence Act), art 86.

7 ‘Proposal for a Regulation of the European Parliament and of the Council on the European Health Data Space’, COM (2022) 197 final, ch II, s 1.

8 Lee A Bygrave, ‘Minding the Machine v2.0: The EU General Data Protection Regulation and Automated Decision-Making’, in Karen Yeung and Martin Lodge (eds), *Algorithmic Regulation* (Oxford University Press 2019) 248–62; Maja Brkan, ‘Do Algorithms Rule the World? Algorithmic Decision-Making and Data Protection in the Framework of the GDPR and Beyond’ (2019) 27 International Journal of Law and Information Technology 91–121.

9 Margot Kaminski, ‘The Right to Explanation, Explained’ (2019) 34 Berkeley Technology Law Journal 189–218; Filip Geburczyk, ‘Automated Administrative Decision-Making under the Influence of the GDPR—Early Reflections and Upcoming Challenges’ (2021) 41 Computer Law & Security Review 105538.

10 Andrew D Selbst and Julia Powles, ‘Meaningful Information and the Right to Explanation’ (2017) 7 International Data Privacy Law 233–42; Sandra Wachter, Brent Mittelstadt, and Luciano Floridi, ‘Why a Right to Explanation of Automated Decision-Making Does Not Exist in the General Data Protection Regulation’ (2017) 1 International Data Privacy Law 76–99.

11 CJEU, Case C–634/21 (*SCHUFA Holding (Scoring)*), ECLI:EU:C:2023:957, 7 December 2023.

12 Eduard Fosch-Villaronga and others, ‘Accounting for Diversity in AI for Medicine’ (2022) 47 Computer Law & Security Review 105735.

13 Kun-Hsing Yu, Andrew L Beam and Isaac S Kohane, ‘Artificial Intelligence in Healthcare’ (2018) 2 Nature Biomedical Engineering 719.

14 Jonathan Cohen and Tamar Ezer, ‘Human Rights in Patient Care: A Theoretical and Practical Framework’ (2013) 2 Health and Human Rights Journal 7–19.

15 Joachim Boldt, ‘The Concept of Vulnerability in Medical Ethics and Philosophy’ (2019) 14 Philosophy, Ethics, and Humanities in Medicine 6.

16 Högberg and Larsson (n 2).

17 Beate Roessler, *The Value of Privacy* (Polity 2005).

18 ‘Patients’ Rights in the European Union: Mapping eXercise : Final report’ (25 January 2018).

19 Van Kolfschooten (n 3).

20 Abdulrahman Takiddin and others, ‘Artificial Intelligence for Skin Cancer Detection: Scoping Review’ (2021) 23 Journal of Medical Internet Research e22934.

21 Owain Jones and others, ‘Artificial Intelligence and Machine Learning Algorithms for Early Detection of Skin Cancer in Community and Primary Care Settings: A Systematic Review’ (2022) 4 The Lancet Digital Health e466.

22 Chiara Corti and others, ‘Artificial Intelligence for Prediction of Treatment Outcomes in Breast Cancer: Systematic Review of Design, Reporting Standards, and Bias’ (2022) 108 Cancer Treatment Reviews 102410.

23 Fang Liu and others, ‘Fully Automated Diagnosis of Anterior Cruciate Ligament Tears on Knee MR Images by Using Deep Learning’ (2019) 1 Radiology: Artificial Intelligence 180091.

24 Ronald Chow and others, ‘Use of Artificial Intelligence for Cancer Clinical Trial Enrollment: A Systematic Review and Meta-Analysis’ (2023) 115 Journal of the National Cancer Institute 365.

25 Fabio Quartieri and others, ‘Artificial Intelligence Augments Detection Accuracy of Cardiac Insertable Cardiac Monitors: Results from a Pilot Prospective Observational Study’ (2022) 3 Cardiovascular Digital Health Journal 201.

26 At the moment, automated closed-loop systems do not provide a full solution. However, the movement of technology-savvy people with type 1 diabetes have been developing open-source ‘Do It Yourself’ systems that enable automated insulin delivery which may further push this development, see eg, Amy E Morrison and others, ‘A Scoping Review of Do-It-Yourself Automated Insulin Delivery System (DIY AID) Use in People with Type 1 Diabetes’ (2022) 17 PLOS ONE e0271096.

27 Sophie Templer, ‘Closed-Loop Insulin Delivery Systems: Past, Present, and Future Directions’ (2022) 13 Frontiers in Endocrinology 919–42.

28 Janice MacLeod and others, ‘Shining the Spotlight on Multiple Daily Insulin Therapy: Real-World Evidence of the InPen Smart Insulin Pen’ (2024) 26 Diabetes TechnolologyTherapy33–39.

29 Pedro J Ballester and Javier Carmona, ‘Artificial Intelligence for the next Generation of Precision Oncology’ (2021) 5 Precision Oncology 1.

30 Eduard Fosch-Villaronga and others, ‘Implementing AI in Healthcare: An Ethical and Legal Analysis Based on Case Studies’ in Dara Hallinan, Ronald Leenes andPaul de Hert (eds) *Data Protection and Artificial Intelligence: Computers, Privacy, and Data Protection* (Hart Publishing 2021) 187–216.

31 Hannah van Kolfschooten, ‘The AI Cycle of Health Inequity and Digital Ageism: Mitigating Biases through the EU Regulatory Framework on Medical Devices’ (2023) 10 Journal of Law and the Biosciences lsad031; Hannah van Kolfschooten and Astrid Pilottin, ‘Reinforcing Stereotypes in Health Care Through Artificial Intelligence–Generated Images: A Call for Regulation’ (2024) 2 Mayo Clinic Proceedings: Digital Health 335.

32 Isabel Straw, ‘The Automation of Bias in Medical Artificial Intelligence (AI): Decoding the Past to Create a Better Future’ (2020) 110 Artificial Intelligence in Medicine 101965.

33 Hannah van Kolfschooten, Pin Lean Lau and Janneke Van Oirschot, ‘AI Can Threaten Health Equity for Marginalised Populations: The EU Must Act Now’ (*Health Action International*, 13 June 2023) <https://haiweb.org/ai-can-threaten-health-equity-for-marginalised-populations/> accessed 1 August 2024.

34 Himabindu Reddy and others, ‘A Critical Review of Global Digital Divide and the Role of Technology in Healthcare’ (2022) 14 Cureus e29739.

35 Jinpei Han and others, ‘A Systematic Review of Robotic Surgery: From Supervised Paradigms to Fully Autonomous Robotic Approaches’ (2022) 18 The International Journal of Medical Robotics and Computer Assisted Surgery e2358.

36 Paul Formosa and others, ‘Medical AI and Human Dignity: Contrasting Perceptions of Human and Artificially Intelligent (AI) Decision Making in Diagnostic and Medical Resource Allocation Contexts’ (2022) 133 Computers in Human Behavior 107296.

37 Luciano Floridi, ‘On Human Dignity as a Foundation for the Right to Privacy’ (2016) 29 Philosophy & Technology 307.

38 Karl Manheim and Lyric Kaplan, ‘Artificial Intelligence: Risks to Privacy and Democracy’ (2019) 21 Yale Journal of Law and Technology 106.

39 Julia Amann and others, ‘Explainability for Artificial Intelligence in Healthcare: A Multidisciplinary Perspective’ (2020) 20 BMC Medical Informatics and Decision Making 310.

40 Line Farah and others, ‘Assessment of Performance, Interpretability, and Explainability in Artificial Intelligence–Based Health Technologies: What Healthcare Stakeholders Need to Know’ (2023) 1 Mayo Clinic Proceedings: Digital Health 120.

41 Marzyeh Ghassemi, Luke Oakden-Rayner and Andrew L Beam, ‘The False Hope of Current Approaches to Explainable Artificial Intelligence in Health Care’ (2021) 3 The Lancet Digital Health e745.

42 Thomas Grote and Philipp Berens, ‘On the Ethics of Algorithmic Decision-Making in Healthcare’ (2020) 46 Journal of Medical Ethics 205.

43 I. Glenn Cohen, ‘Informed Consent and Medical Artificial Intelligence: What to Tell the Patient?’ (2019) 108 Georgetown Law Journal 1425.

44 Ploug and Holm (n 4).

45 ibid.

46 Glenn Cohen (n 43).

47 Colin Mitchell and Corrette Ploem, ‘Legal Challenges for the Implementation of Advanced Clinical Digital Decision Support Systems in Europe’ (2018) 3 Journal of Clinical and Translational Research 424.

48 Ploug and Holm (n 4).

49 Högberg and Larsson (n 2).

50 See, eg, on contract law and tort law: Philipp Hacker and others, ‘Explainable AI under Contract and Tort Law: Legal Incentives and Technical Challenges’ (2020) 28 Artificial Intelligence and Law 415.

51 Johan Hansen and others, *Assessment of the EU Member States’ Rules on Health Data in the Light of GDPR* (European Union 2021) 262.

52 CJEU (n 11).

53 art 15 DPD.

54 art 12(a) and Recital 41 DPD.

55 Lee A Bygrave, ‘Automated Profiling. Minding the Machine: Article 15 of the EC Data Protection Directive and Automated Profiling’ (2001) 17 Computer Law & Security Review 17.

56 COM 422 (1992) 26.

57 Bygrave (n 55).

58 Kaminski (n 9).

59 Paul De Hert and Guillermo Lazcoz Moratinos, ‘Radical Rewriting of Article 22 GDPR on Machine Decisions in the AI Era’ (*European Law Blog* 2021) <https://europeanlawblog.eu/2021/10/13/radical-rewriting-of-article-22-gdpr-on-machine-decisions-in-the-ai-era/> accessed 1 August 2024.

60 CJEU (n 11).

61 art 5(2) GDPR.

62 Guidelines on transparency under Regulation 2016/679, WP260 rev.01, endorsed by EDPB.

63 arts 13(2)(f) and 14(2)(g) GDPR; art 15(1)(h) GDPR.

64 Stefanie Hänold, ‘Profiling and Automated Decision-Making: Legal Implications and Shortcomings’ in Marcelo Corrales, Mark Fenwick and Nikolaus Forgó (eds), *Robotics, AI and the Future of Law* (Springer 2018).

65 CJEU, *SCHUFA Holding (Scoring)* s 43.

66 ibid ss 14–16.

67 ibid ss 45–46. Also, see Recital 71 GDPR.

68 ibid s 46. Also see, Francesca Palmiotto Ettorre, ‘Is Credit Scoring an Automated Decision? – The Opinion of the AG Pikamäe in the Case C-634/21 — The Digital Constitutionalist’ (17 March 2023) <https://digi-con.org/is-credit-scoring-an-automated-decision-the-opinion-of-the-ag-pikamae-in-the-case-c-634-21/> accessed 1 August 2024

69 Advocate General’s Opinion in Case C-634/21 | *SCHUFA Holding and Others (Scoring)* and in Joint Cases C-26/22 and C-64/22 *SCHUFA Holding and Others (Discharge from remaining debts)*, 16 March 2023.

70 art 29 Data Protection Working Party (n 62), p 24.

71 ibid.

72 Alexander M Aroyo and others, ‘Overtrusting Robots: Setting a Research Agenda to Mitigate Overtrust in Automation’ (2021) 12 Paladyn, Journal of Behavioral Robotics 423.

73 Patricia L Hardré, ‘Chapter 5 - When, How, and Why Do We Trust Technology Too Much?’ in Sharon Y Tettegah and Dorothy L Espelage (eds), *Emotions, Technology, and Behaviors* (Academic Press 2016).

74 CJEU, *SCHUFA Holding (Scoring)* s 47. Also, see art 4(4) GDPR.

75 ibid 21.

76 ibid s 48.

77 Max Schemmer and others, ‘On the Influence of Explainable AI on Automation Bias’ [2022] arXiv preprint arXiv:2204.08859.

78 See art 22(2) GDPR.

79 arts 4(11) and recital 32 GDPR.

80 European Data Protection Board, ‘Guidelines 05/2020 on Consent Under Regulation 2016/679’, Version 1.1, Adopted on 4 May 2020, pp 20–22; art 32 GDPR.

81 Onora O’Neill, ‘Some Limits of Informed Consent’ (2003) 29 Journal of Medical Ethics4.

82 Johan Bester, Cristie M Cole and Eric Kodish, ‘The Limits of Informed Consent for an Overwhelmed Patient: Clinicians’ Role in Protecting Patients and Preventing Overwhelm’ (2016) 18 AMA Journal of Ethics 869.

83 See *Lambert and others v France* App no 46043/14 (ECHtR, 5 June 2015) para 74; *Pretty v the United Kingdom* App no 2346/01 (ECtHR, 29 April 2002) para 63; *Trocellier v France* App no 75725/01 (ECtHR, 5 October 2006) para 4; *Y v Turkey* App no 648/10 (ECtHR, 17 February 2015) paras 68–78; *CC v Spain* App no 1425/06 (ECtHR, 6 October 2009) para 33.

84 Andrea Martani and others, ‘The Devil Is in the Details: An Analysis of Patient Rights in Swiss Cancer Registries’ (2022) 48 Journal of Medical Ethics 1048.

85 Griet Verhenneman, ‘Informed Consent, a Means to Empower the Patient?’ in Griet Verhenneman (ed), *The Patient, Data Protection and Changing Healthcare Models: The Impact of e-Health on Informed Consent, Anonymisation and Purpose Limitation* (Intersentia 2021).

86 Gabriela Zanfir, ‘Forgetting About Consent. Why The Focus Should Be On ‘Suitable Safeguards’ in Data Protection Law’ in Serge Gutwirth, Ronald Leenes and Paul De Hert (eds), *Reloading Data Protection: Multidisciplinary Insights and Contemporary Challenges* (Springer 2014).

87 Benjamin Bergemann, ‘The Consent Paradox: Accounting for the Prominent Role of Consent in Data Protection’ in Marit Hansen and others (eds), *Privacy and Identity Management. September 4–8, 2017, Revised Selected Papers* (Springer International Publishing 2018).

88 Glenn Cohen (n 43).

89 Bart W Schermer, Bart Custers and Simone van der Hof, ‘The Crisis of Consent: How Stronger Legal Protection May Lead to Weaker Consent in Data Protection’ (2014) 16 Ethics and Information Technology 171.

90 Riikka Koulu, ‘Proceduralizing Control and Discretion: Human Oversight in Artificial Intelligence Policy’ (2020) 27 Maastricht Journal of European and Comparative Law 720.

91 Riikka Koulu, ‘Human Control over Automation: EU Policy and AI Ethics’ (2020) 12 European Journal of Legal Studies 9.

92 Aurelia Sauerbrei and others, ‘The Impact of Artificial Intelligence on the Person-Centred, Doctor-Patient Relationship: Some Problems and Solutions’ (2023) 23 BMC Medical Informatics and Decision Making 73.

93 Claudio Sarra, ‘Put Dialectics into the Machine: Protection against Automatic-Decision-Making through a Deeper Understanding of Contestability by Design’ (2020) 20 Global Jurist 20200003.

94 Sarah Jabbour and others, ‘Measuring the Impact of AI in the Diagnosis of Hospitalized Patients: A Randomized Clinical Vignette Survey Study’ (2023) 330 Journal of American Medical Association 2275.

95 Ben Green and Amba Kak, ‘The False Comfort of Human Oversight as an Antidote to A.I. Harm’ (*Slate* 2021) <https://slate.com/technology/2021/06/human-oversight-artificial-intelligence-laws.html> accessed 1 August 2024.

96 Eugenio Alberdi and others, ‘Effects of Incorrect Computer-Aided Detection (CAD) Output on Human Decision-Making in Mammography’ (2004) 11 Academic Radiology 909.

97 Thomas Dratsch and others, ‘Automation Bias in Mammography: The Impact of Artificial Intelligence BI-RADS Suggestions on Reader Performance’ (2023) 307 Radiology e222176.

98 Stefan Strauß, ‘Deep Automation Bias: How to Tackle a Wicked Problem of AI?’ (2021) 5 Big Data and Cognitive Computing 18.

99 Saar Alon-Barkat and Madalina Busuioc, ‘Human-AI Interactions in Public Sector Decision-Making: ‘Automation Bias’ and ‘Selective Adherence’ to Algorithmic Advice’ (2022) 33 Journal of Public Administration Research and Theory 153–169.

100 Tina Nguyen, ‘ChatGPT in Medical Education: A Precursor for Automation Bias?’ (2024) 10 JMIR Medical Education e50174.

101 Kristin M Kostick-Quenet and Sara Gerke, ‘AI in the Hands of Imperfect Users’ (2022) 5 npj Digital Medicine 1.

102 Nithya Sambasivan and Rajesh Veeraraghavan, ‘The Deskilling of Domain Expertise in AI Development’, 2022 *Proceedings of the 2022 CHI Conference on Human Factors in Computing Systems* (Association for Computing Machinery).

103 Emanuele Sinagra, Francesca Rossi and Dario Raimondo, ‘Use of Artificial Intelligence in Endoscopic Training: Is Deskilling a Real Fear?’ (2021) 160 Gastroenterology 2212.

104 Grote and Berens (n 42).

105 Fanny Ficuciello and others, ‘Autonomy in Surgical Robots and Its Meaningful Human Control’ (2019) 10 Paladyn, Journal of Behavioral Robotics 30.

106 Dario Amodei and others, ‘Concrete Problems in AI Safety’ (*arXiv.org*, 21 June 2016).

107 Sarra (n 93).

108 ibid.

109 Marco Almada, ‘Human Intervention in Automated Decision-Making: Toward the Construction of Contestable Systems’ (2019) *Proceedings of the Seventeenth International Conference on Artificial Intelligence and Law* (Association for Computing Machinery ).

110 Isak Mendoza and Lee A. Bygrave, ‘The Right Not to be Subject to Automated Decisions Based on Profiling‘in Tatiana Synodinou and others (eds), EU Internet Law (Springer 2017).

111 Almada (n 109).

112 Opinion of Advocate General Cruz Villalon, delivered on 9 July 2015 in Case C-201/14 (*Smaranda Bara and Others*), ECLI:EU:C:2015:461, s 74.

113 Maja Brkan and Grégory Bonnet, ‘Legal and Technical Feasibility of the GDPR’s Quest for Explanation of Algorithmic Decisions: Of Black Boxes, White Boxes and Fata Morganas’ (2020) 11 European Journal of Risk Regulation 18.

114 Wachter, Mittelstadt and Floridi (n 10).

115 Bryce Goodman and Seth Flaxman, ‘European Union Regulations on Algorithmic Decision-Making and a “Right to Explanation”’ (2017) 38 AI Magazine 50.

116 Bryan Casey, Ashkon Farhangi and Roland Vogl (eds), ‘Rethinking Explainable Machines: The GDPR’s Right to Explanation Debate and the Rise of Algorithmic Audits in Enterprise’ (2019) 34Berkeley Technology Law Journal 143–88.

117 Selbst and Powles (n 10).

118 Tiago Sergio Cabral, ‘AI and the Right to Explanation: Three Legal Bases under the GDPR’ in Dara Hallinan, Ronald Leenes and Paul De Hert (eds), *Data Protection and Privacy: Data Protection and Artificial Intelligence* (Bloomsbury Publishing 2021).

119 Stefanie Hänold, ‘Profiling and Automated Decision-Making: Legal Implications and Shortcomings’ in Marcelo Corrales, Mark Fenwick and Nikolaus Forgó (eds), *Robotics, AI and the Future of Law* (Springer 2018).

120 Lilian Edwards and Michael Veale, ‘Slave to the Algorithm? Why a “Right to an Explanation” Is Probably Not the Remedy You Are Looking For’ (2017) 16 Duke Law & Technology Review 18.

